# The Involvement of Bax in Zinc-Induced Mitochondrial Apoptogenesis in Malignant Prostate Cells

**DOI:** 10.1186/1476-4598-7-25

**Published:** 2008-03-10

**Authors:** Pei Feng, Tieluo Li, Zhixin Guan, Renty B Franklin, Leslie C Costello

**Affiliations:** 1 Department of Biomedical Sciences/Dental School and The Greenebaum Cancer Center, University of Maryland, Baltimore, Maryland, USA

## Abstract

**Background:**

The development and progression of prostate cancer requires the transformation of normal zinc-accumulating epithelial cells to malignant cells that have lost the ability to accumulate zinc. This metabolic transformation is essential so that the tumor suppressive effects of zinc can be eliminated and the malignant process can proceed. One of the major effects of zinc is its prevention of prostate cell growth by its induction of apoptosis. The accumulation of cellular zinc has a direct effect on the mitochondria that results in the release of cytochrome c, which initiates the caspase cascade that leads to apoptosis. This effect is associated with the mitochondrial pore-forming process, but the mechanism by which zinc induces the release of cytochrome c and induces mitochondrial apoptogenesis has not been resolved. The present report provides for the first time information that implicates Bax in the zinc induction of mitochondrial apoptogenesis.

**Results:**

The effects of zinc treatment on the Bax levels of PC-3 cells and on the mitochondria were determined. The exposure of isolated mitochondria to zinc results in an increase in membrane bound Bax, which is due to the mitochondrial insertion of endogenous resident Bax. The mitochondrial Bax/Bcl-2 ratio is increased by zinc treatment. Zinc treatment of PC-3 cells also increases the mitochondrial level of Bax. In addition, zinc treatment increases the cellular level of Bax and the cellular Bax/Bcl2 ratio. Down regulation of Bax in PC-3 cells eliminates the zinc induction of apoptosis. The increase in cellular Bax level appears to involve zinc induction of Bax gene expression.

**Conclusion:**

This report extends and confirms that physiological levels of zinc induce apoptosis in prostate cells. The study provides evidence that zinc is directly involved in facilitating a Bax-associated pore formation process that initiates mitochondrial apoptogenesis. This is enhanced by an additional effect of zinc on increasing the cellular level of Bax. To avoid the anti-tumor apoptogenic effects of zinc, the malignant cells in prostate cancer posses genetic/metabolic adaptations that prevent the cellular accumulation of zinc.

## Background

An early event in the development and progression of prostate cancer is the metabolic transformation of normal zinc-accumulating epithelial cells to malignant cells that have lost the ability to accumulate zinc [for reviews see [1-3]]. This metabolic transformation is essential so that the tumor suppressive effects of zinc can be eliminated and the malignant process can proceed. One of the effects of zinc is the prevention of cell growth due to zinc-induced apoptosis that occurs in prostate cells. In previous studies [4-7], we reported that the uptake and accumulation of zinc results in the mitochondrial release of cytochrome c, which initiates the caspase cascade that leads to apoptosis. Moreover, the release of cytochrome c results from a direct and rapid interaction of cytosolic zinc with the mitochondria; presumably through a zinc associated pore-forming process. However, the mechanism by which zinc induces the release of cytochrome c and induces mitochondrial apoptogenesis has not been resolved. We now report that zinc treatment of PC-3 cells facilitates the mitochondrial insertion of Bax (i.e. a pore-forming activity); and also increases the expression and cellular level of Bax.

## Results

### Direct effect of zinc on mitochondrial resident Bax

**Figure 1:**
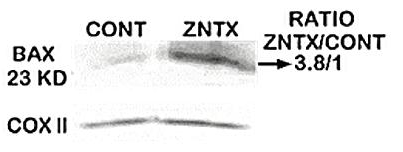
**The direct effect of zinc on the level of bound Bax in isolated mitochondria from PC-3 cells.** The isolated mitochondria were exposed to medium supplemented with 15 μM zinc for 15 minutes or with unsupplemented medium (control).

We previously demonstrated that exposure of isolated prostate mitochondria to zinc results in the release of cytochrome c [6,7]. Because of the role of Bax in the pore-forming process for cytochrome c release [8,9], we determined the direct effect of zinc on the Bax level of isolated mitochondria prepared from PC-3 cells. The mitochondria were exposed to medium supplemented with 15 μM zinc for 15 minutes or with unsupplemented medium (control). The mitochondria were then subjected to alkali treatment to remove weakly bound membrane Bax [9], and assayed for Bax by Western blot. Zinc treatment resulted in a significant 280% increase in mitochondrial-bound Bax (Figure 1). As is the case with the release of cytochrome c [7], the results demonstrate that zinc exhibits a direct effect on the mitochondria that does not require the interaction or involvement of additional cytosolic factors. The results also demonstrate that Bax is a resident protein in the prostate mitochondria; i.e. it exists in mitochondria of cells in the absence of an apoptotic signal.

In some normal mammalian cells Bax is not found as a resident mitochondrial protein [8], but is translocated to the mitochondria from cytosol after the cells are exposed to an apoptosis-inducing signal. Bax insertion into the mitochondrial outer membrane is an initiating pore-forming event in the release of cytochrome c that triggers mitochondrial apoptogenesis [8-11].

### Effect of zinc treatment on the levels of mitochondrial Bax and Bcl-2 in PC-3 cells

**Figure 2:**
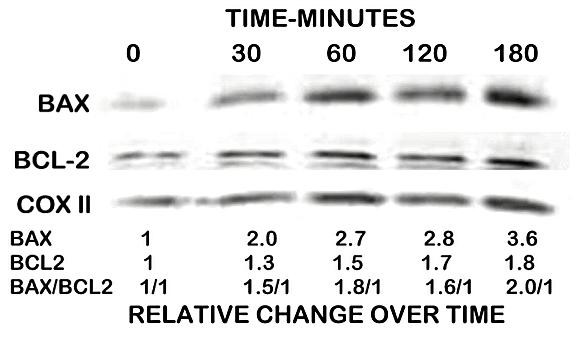
**The effects of zinc on Bax and Bcl-2 levels of mitochondria isolated from PC-3 cells that were exposed to medium supplemented with zinc. **The cells were treated with medium supplemented with 15 μM zinc for 180 minutes versus no zinc treatment.

We then determined if the direct effect of zinc on Bax observed in the isolated mitochondrial preparations also occurred in PC-3 cells that were exposed to zinc-supplemented medium. PC-3 cells were exposed to medium supplemented with 15 μM zinc for 180 minutes vs. no zinc treatment. These are conditions that result in cytochrome c release and pursuant apoptotic events [6,7]. The mitochondria were isolated and the Bax and Bcl-2 levels determined by Western blot. By 30 minutes of exposure to zinc, the mitochondrial level of Bax was increased by about 100%; and further increased by 260% at 180 minutes (Figure 2). Zinc treatment caused a much lower increase in Bcl-2 that ranged from about 30% at 30 minutes to 80% by 180 minutes. Therefore, zinc treatment resulted in a 2-fold increase in the Bax/Bcl-2 ratio. This is a relevant relationship since it is generally recognized that maintenance of an appropriate Bax/Bcl-2 balance in cells prevents apoptosis [8,12]. When this ratio is altered in favor of Bax, cells become apoptotic due to increased translocation of Bax to mitochondria and subsequent release of cytochrome c.

### Effect of zinc treatment on the cellular levels of Bax and Bcl-2 in PC-3 cells

**Figure 3:**
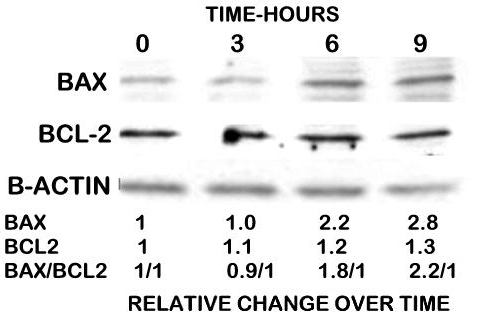
The effect of 15 μM zinc treatment of PC-3 cells on the total cellular levels of Bax and Bcl-2.

The increase in mitochondrial Bax that results from exposure of cells to zinc could be due to the translocation of existing cytosolic Bax and/or an increase in the cellular level of Bax. Therefore we determined the effect of 15 μM zinc treatment on the total cellular levels of Bax and Bcl-2 in PC-3 cells. Figure 3 shows that the cellular level of Bax was unchanged after three hours of zinc treatment; but by six hours the Bax level increased by about 120% and further increased to 180% by nine hours. In contrast, Bcl-2 exhibited only a slight increase of about 30% after nine hours of zinc treatment. Therefore, the zinc treatment caused the relative Bax/Bcl-2 ratio to increase from the normalized pre-treatment ratio of 1/1 to ~2.5/1 following treatment. It is also notable that up to three-hour zinc treatment shown in Figure 3, caused no effect in total cellular Bax; but caused a significant increase in mitochondrial Bax level within 30 minutes that markedly increased by three hours. The rapidity of the increase in mitochondrial Bax that occurs prior to the increase in total cellular Bax demonstrates that zinc has two effects: 1) it facilitates the translocation of existing resident cytosolic Bax to mitochondria; and 2) it facilitates an increase in total cellular Bax level.

**Figure 4:**
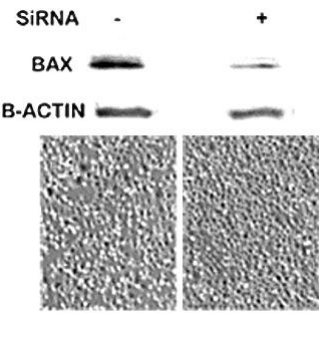
**The effect of zinc on Bax knock-down PC-3 cells.** PC-3 cells were transfected with Bax siRNA or with empty vector and cultured for 72 hours; followed by treatment of the cells with 15 μM zinc for 24 hours.

To corroborate further the linkage of the zinc-Bax-apoptosis relationship, we determined the effect of zinc on Bax knock-down PC-3 cells. PC-3 cells were transfected with Bax siRNA or with empty vector and cultured for 72 hours. This was followed by treatment of the cells with 15 μM zinc for 24 hrs. The cells were then collected for Western blot analyses of Bax and microscopic examination. Figure 4 reveals that siRNA significantly decreased the level of cellular Bax. Zinc treatment induced the expected apoptotic effect in the control cells that expressed Bax; whereas zinc treatment exhibited no apoptotic effect in the Bax-deficient cells.

**Figure 5:**
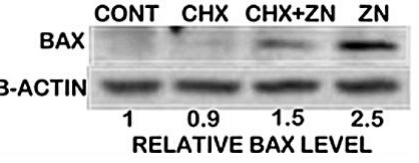
**The effect of cyclohexamide treatment of PC-3 cells on the zinc-induced cellular increase in Bax.** PC-3 cells were cultured in serum-free medium for 24 h and then treated with or without cycloheximide (CHX, 5 μg/ml) for 1 hour prior to zinc (15 μM) exposure for 6 hours.

To obtain some initial insight into the mechanism associated with the increased cellular level of Bax, we determined the effect of cyclohexamide on the zinc-induced increase in Bax. Figure 5 shows that zinc treatment, as expected, increased the cellular level of Bax by about 150%. Cycloheximide treatment resulted in a 67% attenuation of the zinc effect. This provides initial evidence that cellular zinc accumulation might cause an increase in the expression of the Bax gene, which would account for the zinc-induced cellular increase in Bax.

## Discussion

**Figure 6:**
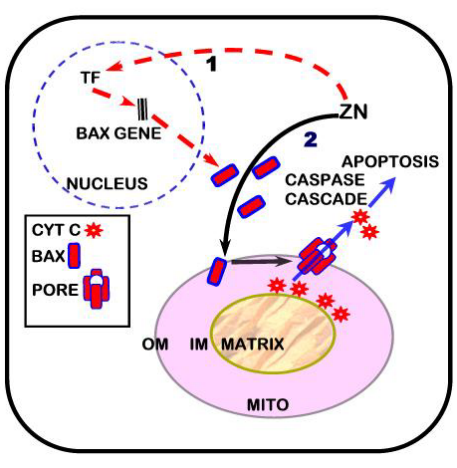
**A representation of the integrated apoptogenic effects of zinc.** Zinc increases the Bax-associated mitochondrial pore forming process that results in the release of cytochrome c that initiates the caspase cascade leading to apoptosis. Zinc also increases the gene expression and cellular production of Bax and the Bax/Bcl-2 ratio, which facilitates the mitochondrial Bax-associated apoptogenic effect.

The results of this study confirm and extend our previous reports [5-7] that zinc induces mitochondrial apoptogenesis in zinc-accumulating prostate cells. It is now evident that zinc exhibits a direct effect on mitochondria that facilitates the insertion of resident Bax, which is involved in the mitochondrial pore-forming process [8,9,11]; and is consistent with the release of cytochrome c that also occurs in response to zinc treatment as we previously reported (Figure 6). Other mechanisms of zinc-associated apoptosis have been reported. For example, zinc-induced Increased ROS production [13] and zinc involvement with NF-kappaB [14] have been suggested to be associated with the induction of apoptosis. These potential alternative mechanisms might be associated with zinc induced apoptosis, but they are not required for or involved with the direct Bax-associated mitochondrial apoptogenic effect that we have identified. The conditions for the preparation of the washed isolated mitochondrial preparations employed in this report and in our earlier reports results in substrate-deficient non-respiring mitochondria that eliminates any ROS production or any involvement of intracellular signaling pathways.

That zinc treatment also induces an increase in cellular Bax levels and in the Bax/Bcl-2 ratio would amplify the zinc effect on mitochondrial Bax (Figure 6). The increase in cellular Bax provides more Bax for translocation to the mitochondria; and the increase in the Bax/Bcl-2 ratio reduces the anti-apoptotic effect of Bcl-2. Zinc induction of an increase in cellular Bax and in the Bax/BCL2 ratio has been observed in other cells [15-17]. However, those reports did not involve studies of direct effects of zinc on mitochondria. It is important to add that we cannot dismiss the possible involvement of other proapoptotic proteins that could be associated with a Bax pore forming process; and such studies are important to pursue.

Although the cause of the increase in cellular Bax levels is yet to be established, the attenuating effect of cyclohexamide indicates a likely stimulatory effect of zinc on Bax gene expression. A search of the promoter region of the Bax gene reveals the absence of consensus MRE sequences. Therefore a direct interaction with Metal-binding Transcription Factor for activation of an MRE is not an available option for an immediate-early Bax gene regulation by zinc. However, several response elements for transcription factors reportedly activated by zinc are present in the Bax promoter [18,19]. Thus mechanisms involving zinc induction of transcription factors with subsequent induction of Bax expression do exist and need to be investigated.

The apoptogenic effect of zinc has important implications in regard to prostate cancer [1-3]. The normal prostate glandular epithelial cells evolved for the unique specialized capability and function of high zinc accumulation. However, high cellular zinc levels have potential adverse consequences on cellular activity: such as the metabolic/bioenergetic effects of its inhibition of citrate oxidation and truncation of the Krebs cycle; and the induction of apoptosis. The normal prostate glandular epithelial cells survive and function under these conditions due to their incorporation and adaptation of conditions and activities that compensate and modulate the potential adverse effects of zinc. For example, the metabolic/bioenergetic consequences are compensated by the incorporation of a high aerobic glycolysis and an aspartate-glutamate-citrate pathway, which normally do not exist in most mammalian cells. In the case of the apoptotic effect, the normal cells likely invoke anti-apoptotic activities. For example, HIF-1a expression reportedly prevents the apoptotic effect of zinc in normal prostate glands [13]. Our identification of the role of Bax would suggest that the normal prostate glandular epithelial cells likely incorporate upregulation of anti-apoptotic proteins such as Bcl-2 that prevent the mitochondrial apoptogenic effects of zinc. This is an important issue and relationship that needs to be pursued

Since the malignant cells are not functional specialized glandular epithelial cells (they are parasitic cells), they would not be expected to retain the specialized function and capability of zinc accumulation; nor retain the protective mechanisms that prevent the adverse effects of zinc. Consistent with this, it is now established that the high zinc levels that characterize normal human glandular epithelial cells are dramatically decreased in the epithelium of adenocarcinomatous glands. The malignant cells in situ lose the ability to accumulate zinc due to the down regulation of the zinc uptake transporters, especially ZIP1 [20,21]. This releases the malignant cell from the apoptotic effects of zinc; thereby allowing the growth/proliferation of malignancy. In the presence of the normally high cellular zinc levels, the malignant cells would remain in an arrested pre-malignant stage.

Unlike the in situ malignant prostate cells, PC-3 cells and other malignant prostate cell lines do express ZIP1 and the ability to accumulate zinc. When employed in the xenograft tumorigenic model, zinc treatment of the animals results in an inhibition of tumor growth which is accompanied by an increase in the tumor cell zinc accumulation, an increase in apoptosis, and an increase in Bax level and Bax/Bcl-2 ratio [22]. This provides evidence that the in vitro apoptogenic effects of zinc are physiological as well as clinical relevant effects of zinc. It is also notable that zinc treatment exhibits apoptotic anti-tumor effects in other cells [3,15-17,23]. These recent reports and the effects of zinc on the cellular levels of Bax and induction of apoptosis reported here support our proposal that zinc is an tumor suppressor agent in prostate cancer and a potential therapeutic agent for prostate cancer.

## Conclusion

This report extends and confirms that zinc induces apoptosis in prostate cells. The study provides evidence that zinc is directly involved in facilitating a Bax-associated pore formation process. This provides a mechanism for the release of cytochrome c [7], which initiates the caspase cascade that leads to apoptosis. This effect is enhanced by an additional action of zinc on increasing the cellular level of Bax, possibly through an increase in the Bax gene expression. The coordination of these effects is represented in Figure 6. In order to prevent the anti-tumor apoptogenic effects of zinc, the malignant cells in situ in prostate cancer invoke genetic/metabolic transformations that prevent the cellular accumulation of zinc.

## Methods

### PC-3 Cell Culture and Zinc Treatment

The PC-3 malignant prostate cell line was obtained from American Type Culture Collection (ATCC, Rockville, MD), and were maintained in RPMI-1640 medium with glutamine, and supplemented with 10% fetal bovine serum (FBS) and 1% penicillin/streptomycin at 37°C in a humidified atmosphere with 5% CO_2_. Generally, 24 h before the treatment with zinc (15 μM), the growth medium was replaced with serum free medium. The cells were treated with zinc for variable times as indicated in the Results. The cells were then used for isolation of mitochondria and/or preparation of total cellular protein. The isolated mitochondria were subject for alkaline extraction.

### Transfection of SIRNA of Bax to knock down the cellular Bax level

PC-3 cells were transfected with scrambled (control) or with Bax siRNA (100 pmols/ml, Santa Cruz Biotech) and cultured in growth medium for 48 h. Following another 24 h culture in serum-free medium, the cells were then treated without or with zinc for 24 h. Cell proliferation and apoptotic characteristics were examined under light microscopy and the cellular level of Bax was determined by Western Blot analyses.

### Mitochondrial Preparation and zinc treatment of isolated mitochondria

PC-3 cells were harvested, and the mitochondria were isolated under conditions as described previously [7]. The protein concentration of the mitochondrial suspension was measured by the method of Bradford [24]. Aliquots of the suspension (100 μg of protein) were then were employed in the reactions in the absence and presence of added zinc (10 ng/μg mitochondrial protein) at 30°C for 15 min. At the end of the incubation, the mitochondria were collected by rapid centrifugation at 10,000 × g for 5 min, and the mitochondrial pellets were then subjected to alkaline extraction.

### Alkali extraction of mitochondria and detection of Bax, Bcl-2 by Western Blot

For alkaline extraction, the mitochondrial pellets were re-suspended (1 mg protein/ml) in freshly prepared 0.1 M Na_2_CO_3 _(pH 11.5) and incubated for 20 min at 4°C [25]. The mitochondrial membranes were then collected by centrifugation at 360,000 × g for 20 min at 4°C. The levels of Bax and Bcl-2 in the samples were detected by Western blot analyses as described previously [6]. Western blot assays were conducted with specific antibodies for human Bax (Santa Cruz Biotech) and for human Bcl-2 (BDBiosciences Pharmingen). The specificity of Bax signals recognized by the antibody was further confirmed by the pre-blocking experiment using Bax antibody. The amount of total cellular protein loaded for each sample was monitored by the signals for β-actin used as an internal control; mitochondrial protein CoxII was employed as the internal control to adjust the loading amount of the mitochondrial samples. The intensities of specific bands were identified and recorded with an AlphaImager™2000 (Alpha InnotechCorp.). All experiments reported herein were repeated at least three times.

## Competing interests

The author(s) declare that they have no competing interests.

## Authors' contributions

PF, RF, LC conceived the study, participated in its design and coordination, reviewed the data and the application of statistics, and helped to draft the manuscript. TL and ZG set up and conducted experiments and performed assays. All authors read and approved the final manuscript.
